# Multicomponent Lipid Nanoparticles as a Tool to Potentially Improve the Antibiofilm Activity of Resveratrol against MDR Gram-Positive and Gram-Negative Clinical Isolates

**DOI:** 10.1021/acsomega.5c12649

**Published:** 2026-04-24

**Authors:** Giulia Di Prima, Maria Rita Tricoli, Nicola Serra, Viviana De Caro, Ignazio Arrigo, Cecilia La Mantia, Paola Di Carlo, Orazia Diquattro, Anna Giammanco, Teresa Fasciana

**Affiliations:** † Department of Biological, Chemical and Pharmaceutical Sciences and Technologies, 18998 University of Palermo, Via Archirafi 32, 90123 Palermo, Italy; ‡ Department of Health Promotion, Mother and Child Care, Internal Medicine and Medical Specialties G. D’Alessandro, University of Palermo, Piazza Delle Cliniche, 2, 90127 Palermo, Italy; § Audiology Unit, Department of Neuroscience, Reproductive Sciences and Dentistry, 9307 University of Naples Federico II, Via Pansini 5, 80131 Naples, Italy; ∥ Department of Precision Medicine in Medical, Surgical and Critical Care (Me.Pre.C.C.), University of Palermo, Via Liborio Giuffrè, 5, 90127 Palermo, Italy; ⊥ Laboratory of Microbiology, A. O. Ospedali Riuniti “Villa Sofia-Cervello”, 90100 Palermo, Italy

## Abstract

Background: Among natural compounds suggested as antimicrobials to overcome the increasing phenomenon of multidrug resistance (MDR), resveratrol (3,5,4′-trihydroxystilbene; RSV), a phytoalexin commonly found in food and drinks, can be considered a valid alternative. Despite its potentially useful antimicrobial action, orally administered RSV fails in efficacy due to several drawbacks (e.g., first pass effect metabolism, instability, poor water solubility), whereas targeted administration could potentially allow efficacious RSV concentrations against infectious diseases. Additionally, RSV clinical use today is restricted due to low stability and solubility, issues that can be overcome by embedding RSV into lipid-based nanocarriers. Recently, ad hoc designed multicomponent lipid nanoparticles loaded with RSV (mLNP-RSV) were optimized and characterized, showing promising preliminary antibiofilm properties. Methods: In this work, the mLNP-RSV was deeply investigated to assess its potential use for the therapy of Gram-positive and Gram-negative MDR infections. Their antibiofilm action against ATCC 12972 *Staphylococcus aureus* and ATCC 27853 *Pseudomonas aeruginosa* strains was examined in terms of inhibition of biofilm production. Then, the assay was conducted again against clinical strains of *P. aeruginosa* and *S. aureus* isolated from blood samples of hospitalized patients. Results: The administration of free RSV 16 μg/mL determined a biofilm production inhibition of 35.6% and 62.1% for ATCC 12972 *S. aureus* and ATCC 27853 *P. aeruginosa*, respectively, which was further enhanced by administering an equal RSV dose through mLNP-RSV: 51.4% and 63.0% for *S. aureus* and *P. aeruginosa*, respectively. The performed statistical analyses of the data collected from the clinical isolates confirmed the ability of the mLNP-RSV to strongly reduce the formation of a biofilm, especially in *P. aeruginosa* strains. Conclusions: These findings suggest the potential of the proposed nanosystem to address RSV limitations while supporting its therapeutic action, indicating that mLNP-RSV could serve as a promising tool for the treatment of MDR infections.

## Graphical abstract

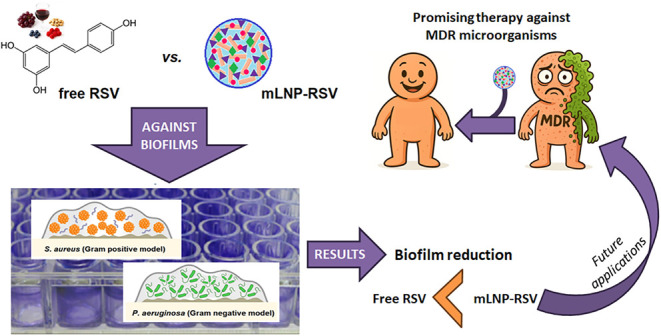

## 1 Introduction

During the last 10 years, many natural compounds have been proposed as antibacterial agents against Gram-positive and Gram-negative multidrug-resistant (MDR) strains. Among these, mushroom extracts have shown particularly promising results due to their high content of bioactive compounds with antimicrobial properties against *S. aureus*.^1^

Recently, Resveratrol (3,5,4′-trihydroxystilbene, RSV) has emerged as a potential antimicrobial agent. RSV is a natural phytochemical compound present in several plants and is commonly found in products for human consumption, such as red wine and grapes. It is considered safe for human consumption and is also employed as a food preservative.^2,3^ Importantly, RSV exhibits antibacterial and antibiofilm activities against both Gram-positive and Gram-negative bacteria.^4,5^ At subinhibitory concentrations, RSV has been shown to reduce bacterial motility, interfere with quorum sensing (QS), reduce toxin production by bacteria, and inhibit biofilm formation.^6^

Biofilm formation is widely recognized as one of the main mechanisms contributing to the development of multidrug resistance in bacteria. Nowadays, increasing attention has been directed at the inhibitory effects of natural compounds, such as RSV, on bacterial biofilm formation. These compounds have been proposed as promising alternatives for the treatment of bacterial infections for their ability to suppress different stages of biofilm formation and/or QS network inhibition.^7,8^

Unfortunately, the clinical application of RSV is restricted by its low solubility in aqueous media, light instability, and low bacterial affinity. These limitations can be effectively addressed by developing suitable drug delivery systems (DDSs). This approach has already been explored using liposomes as delivery systems.^9^ In particular, RSV has been incorporated into cationic liposomes able to enhance its water solubility and stability, prevent its degradation, and promote its interaction with bacterial biofilms.^10^ Despite these advantages, liposomes, which have been the first investigated drug carriers, might suffer from physical and chemical instability, exhibiting limited drug loading capacity, high production costs, and significant variability.^11^ As a consequence, over the past decade, lipid-based nanoparticles have been developed to overcome the limitations of liposomes and have emerged as effective alternative carriers due to their biomimetic properties, biocompatibility, and ability to overcome the physical, chemical, and biological barriers produced by bacteria. Notably, they show significant potential against Gram-positive bacteria by enhancing cell membrane permeability to drugs^12,13^ and Gram-negative pathogens by facilitating polysaccharide disruption, destabilizing membranes, and enabling the release of encapsulated drugs.^14^

Lipid-based nanoparticles, defined as being composed exclusively of lipids, can be classified into solid lipid nanoparticles (SLNs—consisting of at least one solid lipid), nanostructured lipid carriers (NLC—composed of at least two lipids: one solid and one liquid), and multicomponent lipid nanoparticles (mLNPs). The latter represent an evolution of NLCs, as they combine solid and liquid lipids with the addition of other functional lipid components, which could synergize the drug action and/or confer further properties to the DDSs.^15,16^

Given the increasing emergence of multidrug-resistant strains associated with acute and chronic infections due to the inappropriate and excessive use of antibiotics, the administration of natural antimicrobials through innovative lipid-based DDSs represents a promising strategy to effectively prevent the onset and progression of bacterial resistance by limiting biofilm formation. Specifically, Di Prima et al. recently optimized a mLNP-RSV formulation, which demonstrated promising antibiofilm activity at extremely low concentrations.^16^ Based on these encouraging results, this study aims to evaluate the ability of mLNP-RSV to inhibit biofilm formation in comparison with free RSV applied at subinhibitory concentrations against clinical isolates of*P. aeruginosa*and*S. aureus*.

## 2 Materials and Methods

### 2.1 Chemicals

Trans-Resveratrol (RSV) and 18-β-glycyrrhetinic acid were purchased from A.C.E.F spa (Fiorenzuola D’Arda, Piacenza, Italy). Pluronic F-127 was supplied by Sigma-Aldrich (Milan, Italy). Glyceryl monostearate 55–60 was obtained from Farmalabor (Canosa di Puglia, Italy). Labrasol was kindly supplied by Gattefossé (Lyon, France). Menthol was purchased from Carlo Erba Reagents (Milan, Italy). Trifluoroacetic acid (TFA) was obtained from Merck (Darmstadt, Germany). The citrate buffer (pH 5.5) was prepared by dissolving 2.052 g of sodium citrate dihydrate and 0.636 g of citric acid monohydrate in 1 L of distilled water. All chemicals and solvents (analytical grade) were purchased from Carlo Erba Reagents (Milan, Italy) and were used without any further purification.

### 2.2 mLNP-RSV Preparation and Characterization

The RSV-loaded mLNPs (mLNP-RSV) were prepared and characterized according to the previously reported methods.^15,16^

#### 2.2.1 Lipid Mixture Preparation

2 g of lipid mixture was prepared according to the percentage composition reported in Table 1. To prepare the lipid mixture, RSV was dissolved in Labrasol at 40.0 ± 0.5 °C, and then the temperature was increased up to 60.0 ± 0.5 °C and the other components were added and melted. Finally, a clear solution was obtained and then cooled until solidification.

**Table 1:** Percentage Composition (w/w) of the Lipid Mixture

components	percentage Amount (w/w)
Labrasol	30
Glyceryl monostearate	60
18-β-Glycyrrhetinic acid	3
Menthol	2
Trans-Resveratrol	5

RSV integrity throughout the process and its quantification (named RSV_MIX_) were determined by HPLC–DAD analysis, according to a method previously described in the literature^15^ and performed in triplicate on the three prepared batches of lipid mixture. Results are reported as mean (*n* = 9) ± SE.

### 2.2.2 mLNP-RSV Preparation

300 mg of the lipid mixture were melted and emulsified with 40 mL of preheated (70.0 ± 0.5 °C) citrate buffer pH 5.5 containing 400 mg of Pluronic F-127 by using a homogenizer (Kinematica Polytron Model PT MR 2100, Kinematica AG, Malters, Switzerland) at 19,000 rpm for 1 min. The hot coarse emulsion was then subjected to two cycles of high-frequency sonication (frequency: 20 kHz; amplitude: 88–90%; pulse condition: 0.7 s of activity and 0.3 s of inactivity for a total of 10 min) by using an ultrasonic homogenizer (Sonopuls, Bandelin, model HD 2070, Berlin, Germany). This procedure was repeated twice, first at room temperature and subsequently by placing the dispersion into an ice/water/NaCl bath to allow mLNP-RSV solidification.^16^

### 2.2.3 mLNP-RSV Characterization

After each preparation, the quality of the resulting mLNP-RSV was assured by RSV quantification and DLS analyses. The latter were performed at 25.0 ± 0.5 °C using a Malvern Zetasizer NanoZS instrument (Malvern, Worcestershire, U.K.) (λ_laser_ = 532 nm; fixed scattering angle = 173°). The particles’ diameter and polydispersity index (PDI), obtained through cumulative analyses of the correlation function, were considered. Furthermore, the Z-potential was also evaluated.^17^ Results are reported as mean (*n* = 9) ± SE.

The quantitative assessment of RSV into the mLNP-RSV was performed indirectly, as previously reported.^16^ Briefly, following appropriate dilution with citrate buffer at pH 5.5, an aliquot of the resulting dispersion was placed into a filter tube equipped with an inert porous membrane (Ultrafree-MC, Millipore, Burlington, MA; molecular weight cutoff 30,000 Da) and subjected to centrifugation for 20 min at 4000 rpm. The filtrate collected in the lower chamber was analyzed by HPLC-DAD, as described below, to determine the amount of nonencapsulated RSV (RSV_OUT_). Additionally, the total RSV recovered (RSV_TOT_) into the whole dispersion (RSV loaded into the mLNP-RSV + free RSV) was quantified by diluting the dispersion with methanol and analyzing the resulting solution by HPLC-DAD. Consequently, the results were expressed as Drug Recovery% (DR%), representing the total RSV recovered in the mLNP-RSV dispersion relative to the theoretical RSV content in the lipid mixture, Drug Loading% (DL%), corresponding to the amount of RSV encapsulated as a function of the lipid mixture employed, and Loading Efficacy% (LE%), indicating the encapsulated RSV with respect to the total RSV recovered, according to the following equations

$$DR\;\%=\frac{{RSV}_{TOT}\;\left(mg\right)}{{RSV}_{MIX}\;\left(mg\right)}\times 100$$

$$DL\;\;\%=\frac{{RSV}_{TOT}-{RSV}_{OUT}\;\left(mg\right)}{lipid\;mixture\;\left(mg\right)}\times 100$$

$$LE\;\;\%=\frac{{RSV}_{TOT}-{RSV}_{OUT}\;\left(mg\right)}{{RSV}_{TOT}}\times 100$$

The results are reported as mean (*n* = 9) ± SE.

### 2.3 Bacterial Isolates and Growth Conditions

The clinical strains of *P. aeruginosa* (*n* = 50) and *S. aureus* (*n* = 50) were isolated from blood samples withdrawn for routine analysis by hospitalized patients at the Azienda Ospedaliera Universitaria Policlinico “P. Giaccone” of Palermo. Therefore, all data used in the study were anonymized, according to the requirements set by the Italian Data Protection Code (leg. Decree 196/2003), and the general authorizations issued by the Data Protection Authority. Approval by the Ethics Committee was obtained from Azienda Ospedaliera Universitaria Policlinico “P. Giaccone” of Palermo (protocols n◦07/2019).

The strains′ identifications and susceptibility tests were performed by BD Phoenix (Becton Dickinson Europe Holdings SAS-Francia, Pont-de-Claix, France). All antimicrobial susceptibility testing data were interpreted according to EUCAST clinical breakpoints (Supporting Information, Tables S1 and S2). Isolates were cultured on Tryptone Soy Agar (TSA) for routine maintenance and storage. The bacterial strain ATCC 12972 (*S. aureus*) and ATCC 27853 (*P. aeruginosa*) were used as control.^1^

### 2.4 Determination of Biofilm Formation

Before determining the activity against biofilm in clinical isolates, the minimum inhibitory concentration in biofilm formation was evaluated using the broth microdilution method on brain heart infusion (BHI) for two control bacterial strains ATCC 12972 (*S. aureus*) and ATCC 27853 (*P. aeruginosa*).^18^ To determine the lowest RSV concentration able to inhibit biofilm formation, the Crystal violet assay (CVA) was performed by using several RSV concentrations (2, 4, 8, 16, 32, and 64 μg/mL) both as a solution (in DMSO) and as mLNP-RSV dispersions. As controls, empty mLNP (mLNP-BL) diluted according to the mLNP-RSV samples and DMSO dilutions corresponding to free RSV solutions were tested. The latter, which showed no activity at any concentration, are not reported.

The bacterial solution concentration (for both control bacterial strains and clinical strains) was adjusted to 1 × 10^6^ CFU/mL, and 100 μL of bacterial solution was inoculated per well in a 96-well culture plate.

For evaluation on clinical strains, mLNP-RSV or free RSV were added at an RSV concentration equal to 16 μg/mL.

CVA was executed to quantify the biofilm formation. Briefly, 20 μL per well of *S. aureus* and *P. aeruginosa* culture (0.5 McFarland) were inserted into a 96-well plate and then added with

- 180 μL of BHI (control group – CG);
- 178 μL of BHI plus 2 μL of Trans-Resveratrol solutions (free RSV group);
- 160 μL of BHI plus 20 μL of mLNP-RSV dispersions (mLNP-RSV group);

and incubated at 37 °C for 24 h. The blank control was BHI broth, and each well was repeated three times as parallel samples. The planktonic bacteria were washed off using sterile phosphate-buffered saline (PBS). Each well was fixed with 200 μL of methanol for 15 min and dried at room temperature after removing the methanol. The formed biofilms were stained using 200 μL of crystal violet for 20 min. After removing the crystal violet, each well was washed 3 times with PBS. Then, 200 μL of acetic acid solution (33% v/v) was added to each well and incubated for 30 min. The optical density (OD) of the resulting solutions was measured at 570 nm using a microtiter plate reader (Multiskan Go, Thermo Fisher Scientific, Waltham, MA, USA). The selected strains were classified as strong biofilm producers based on the cutoff OD (ODc). The latter was established by evaluating 3 standard deviations above the mean OD of the untreated control. The strain was then classified as follows: OD ≤ ODc = no biofilm producer, ODc < OD ≤ (2 × ODc) = Weak biofilm producer, 2 ODc < OD ≤ (4 × ODc) = Moderate biofilm producer, (4 × ODc) < OD = Strong biofilm producer.^18^

The inhibitory activity of biofilm production, after each treatment, was expressed as the percentage of biofilm reduction according to the following equation

$$\mathrm{bio}\;\mathrm{film}\;\mathrm{reduction}\;\%=\frac{(\mathrm{control}\;\mathrm{group})-\left(\mathrm{processing}\;\mathrm{group}\right)}{\left(\mathrm{control}\;\mathrm{group}\right)}\times 100$$

### 2.5 PCR for the Detection of Genes Involved in Biofilm Formation

Isolates were screened for the presence of some specific genes involved in biofilm formation by PCR amplification using the primers reported in Table 2.

**Table 2:** Primer Sequence, Annealing Temperature, and Amplified Length (bp) for PCR of Genes Involved in Biofilm Formation in *S. aureus* and *P. aeruginosa*

gene in *S. aureus*	primer sequence (5′ → 3′)	amplicon Size	annealing temperature	reference
*icaA*	ACACTTGCTGGCGCAGTCAA	188	55 °C	Mack et al. (2001)^19^
	TCTGGAACCAACATCCAACA			
*icaD*	ATGGTCAAGCCCAGACAGAG	198	55 °C	Rohde et al. (2001)^20^
	AGTATTTTCAATGTTTAAAGCAA			
*clfA*	ATTGGCGTGGCTTCAGTGCT	288	55 °C	Tristan et al. (2003)^21^
	CGTTTCTTCCGTAGTTGCATTTG			
*clfB*	CACTTACTTTACCGCTACTTTC	968	55 °C	Rohde et al. (2001)^20^
	AACGAGCAATACCACTACAACAG			
*fnbpA*	ACCGTCAAACGCAACACAAG	259	55 °C	O’Neill et al. (2008)^22^
	TTCTGATGCCGTTCTTGGCT			
*fnbpB*	GTAACAGCTAATGGTCGAATTGATACT	523	55 °C	Pietrocola et al. (2019)^23^
	CAAGTTCGATAGGAGTACTATGTTC			
*algD*	CTACATCGAGACCGTCTGCC	593	58 °C	Banar M. et al. (2016)^24^
	GCATCAACGAACCGAGCATC			
*pelF*	GAGGTCAGCTACATCCGTCG	789	58 °C	Banar M. et al. (2016)^24^
	TCATGCAATCTCCGTGGCTT			
*pslD*	TGTACACCGTGCTCAACGAC	369	56 °C	Banar M. et al. (2016)^24^
	CTTCCGGCCCGATCTTCATC			

In brief, the DNA was extracted by using the QIAamp DNA Mini Kit (QIAGEN) and all amplifications were carried out on a GeneAmp-9700 (Applied Biosystems) in the following conditions: initial 5 min denaturation at 94 °C followed by 35 cycles of 30 s denaturation at 94 °C, 30 s annealing at the corresponding temperature of the specific pair of primers used and 1 min extension at 72 °C, with a final extension at 72 °C for 7 min. PCR products were analyzed by agarose gel electrophoresis (2.5% agarose in Tris-borate EDTA) in the presence of GelRed (0.3 μg/mL), and the gel images were captured on a gel documentation system (GelDoc, Bio-Rad).

### 2.6 Statistical Analysis

Statistical analyses were performed using the Matrix Laboratory (MATLAB) analytical toolbox version 2008 (MathWorks, Natick, MA). Data are presented as number and percentage for categorical variables, and numerical data are expressed as the mean ± standard deviation (SD) or median with Interquartile range (IRQ = [Q1, Q3]). For CG, free RSV and mLNP-RSV groups, the OD scores and the category were defined for all isolates. Particularly, the categories were defined according to the following classification: Absent (OD ≤ 0.078) as category 1; Weak (0.078 < OD ≤ 0.156) as category 2; Moderate (0.156 < OD ≤ 0.31) as category 3; and Strong (OD > 0.31) as category 4. Additionally, the Global category variable was defined assigning the following scale: category 1 = 1, category 2 = 2, category 3 = 3, and category 4 = 4.

Chi-square test and Fisher’s exact test were performed to evaluate significant differences in proportions or percentages between the two groups. The multiple-comparison chi-square or Fisher’s exact test was used to define significant differences among three or more percentages for unpaired data. If the chi-square or Fisher’s exact test were significant (*p*-value <0.05), the post hoc test was performed using the adjusted standardized residuals and the Z-test. Fisher’s exact test was used where the chi-square test was not appropriate. McNemar’s exact test was used to test the difference between paired proportions.

Cochran’s Q tests were used to compare the differences among percentages for paired data, considering the null hypothesis that there are no differences between the variables or modalities. When Cochran’s Q test was positive (*p*-value <0.05), a minimum required difference for a significant difference between two proportions was calculated using the minimum required differences method with Bonferroni *p*-value corrected for multiple comparisons.

The normality test was performed by the Shapiro-Wilk test. In this case, with a *p*-value <0.05, the hypothesis of data normality was rejected.

The *t* test was used to test the differences between two means of unpaired data. Alternatively, the Mann–Whitney test was used when the distribution was not normal. The Friedman ANOVA test was used to test the difference between several samples when on the same sample, the response to different treatments was evaluated and used as an alternative to Repeated measures analysis of variance for non-normal data distribution. When the Friedman test was positive (*p* < 0.05), the post hoc Wilcoxon test for pairwise comparison was performed. Particularly, where the tests on medians showed a significant difference and the medians were equal, then the mean rank values were specified.

A Kruskal–Wallis test, followed by Conover post hoc pairwise comparisons, was performed to assess multiple comparisons among mLNP-RSV, RSV-free, and mLNP-BL at 2, 4, 8, 16, 32, and 64 μg/mL under conditions of non-normal data distribution. In cases where the Kruskal–Wallis test produced nonsignificant results, post hoc Conover’s test for pairwise comparisons was not performed to avoid inflating the Type I error rate.

Finally, all *p*-values were always two-sided and all tests with *p*-value (*p*) < 0.05 were considered significant.

## 3 Results

Despite RSV’s broad pharmacological potential and natural abundance, its clinical application is limited by unfavorable physicochemical properties, instability, poor water solubility, and low oral bioavailability. Encapsulation into lipid nanoparticles represents an effective strategy to overcome these limitations by enhancing stability, bioavailability, and controlled release. The rational design of the proposed mLNP-RSV resulted in a promising nanosystem exhibiting strong antioxidant activity, along with notable wound-healing properties, confirmed by a fibroblast scratch assay previously demonstrated.^16^ For this study, freshly prepared mLNP-RSV were obtained using a previously optimized hot-melt, high-energy top-down method. To validate the quality of the prepared batch in comparison with the previously optimized formulation, key physicochemical parameters defining the nanodispersion, namely, average particle size, polydispersity index (PDI), and zeta potential, were determined, together with the parameters describing the efficacy of the RSV nanoencapsulation process, namely, Drug Recovery% (DR%), Drug Loading% (DL%), and Loading Efficacy% (LE%). The resulting nanoparticles showed values (Table 3) consistent with those reported in previously published studies.^15,16^

**Table 3:** Characteristics of the mLNP-RSV^a^

parameter	value
DR%_RSV_	98.40 ± 0.55%
DL%_RSV_	4.70 ± 0.02%
LE%_RSV_	95.52 ± 0.03%
Diameter	168.10 ± 3.13 nm
PDI	0.250 ± 0.020
Z-potential	–21.91 ± 5.55 mV

a Mean (n = 9) ± SE

Therefore, these nanoparticles were used to test their antibiofilm properties.

Since the primary objective of this study was not to investigate the bacteriostatic or bactericidal effects of mLNP-RSV, but rather to test the hypothesis that mLNP-RSV can inhibit biofilm formation at subinhibitory concentrations, a pilot experiment was conducted using the reference strains *S. aureus* ATCC 12972 and *P. aeruginosa* ATCC 27853. For this purpose, RSV concentrations of 2, 4, 8, 16, 32, and 64 μg/mL were evaluated as both free RSV solutions and mLNP-RSV dispersions. All concentrations tested were below the minimum inhibitory concentration (MIC) values reported in the literature for RSV against *S. aureus* and *P. aeruginosa* (100–1000 μg/mL and 400–1000 μg/mL, respectively),^25^ indicating that the observed effects were not attributable to bacteriostatic or bactericidal activity. Moreover, to validate the efficacy of RSV-loaded mLNP, empty mLNP (mLNP-BL) diluted according to the mLNP-RSV samples were tested.

The experimental results shown in Figure 1 are expressed as mean percentages of biofilm reduction, calculated from three technical measurements for each solution tested at 2, 4, 8, 16, 32, and 64 μg/mL. At 2, 8, 16, and 64 μg/mL, no significant differences in mean biofilm reduction were observed among mLNP-RSV, RSV-free, and mLNP-BL for either *S. aureus* or *P. aeruginosa*. These findings indicate a comparable effect of free RSV, mLNP-BL, and mLNP-RSV on the biofilm reduction in ATCC strains at these concentrations. In contrast, at 4 and 32 μg/mL, significant differences were detected only for *P. aeruginosa* using the Kruskal–Wallis test (*p* = 0.039 for both). Specifically, the post hoc Conover test showed that at 4 μg/mL the highest percentage of biofilm reduction was observed for mLNP-RSV compared with free RSV (53.01% vs 32.49%, *p* < 0.05) and with mLNP-BL (53.01% vs 26.55%, *p* < 0.05), whereas no significant difference was found between free RSV and mLNP-BL (32.49% vs 26.55%, *p* > 0.05). At 32 μg/mL, biofilm reduction was higher for mLNP-RSV compared with mLNP-BL (62.61% vs 31.34%, *p* < 0.05), and for free RSV compared with mLNP-BL (58.89% vs 31.34%, *p* < 0.05), while no significant difference was observed between mLNP-RSV and free RSV (62.61% vs 58.89%, *p* > 0.05).

**Figure 1:**
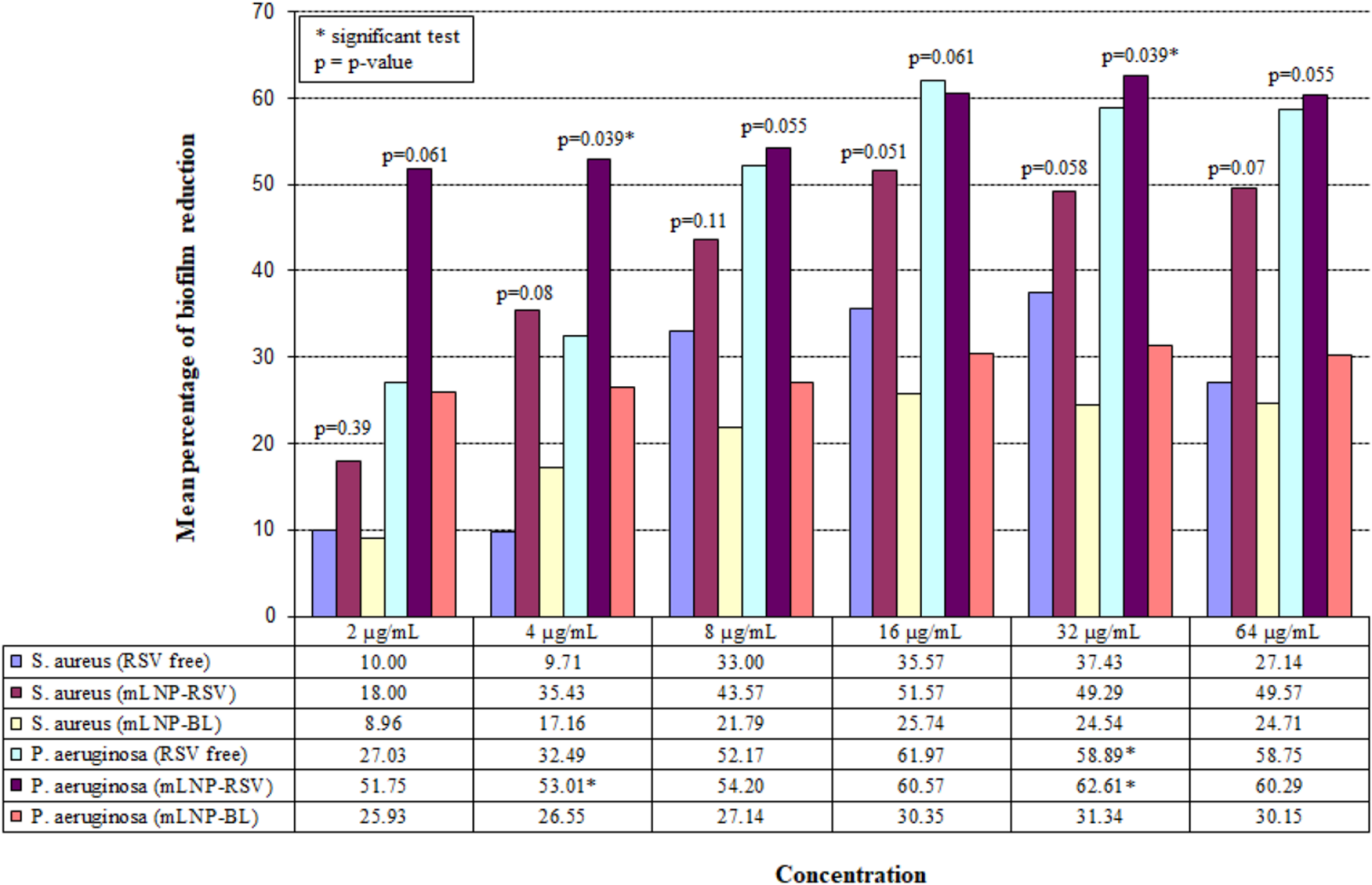
Mean percentage of biofilm reduction in ATCC strains treated with free RSV, mLNP-BL, and mLNP-RSV at different concentrations. The *p*-values shown in the figure refer to the Kruskal–Wallis tests.

As the concentration selected for subsequent testing needed to ensure efficacy against both *S. aureus* and *P. aeruginosa*, the ratios of the mean percentage reduction between the two species were evaluated at 2, 4, 8, 16, 32, and 64 μg/mL (Figure 2). Figure 2 indicates that the optimal ratio occurs at 16 μg/mL.

**Figure 2:**
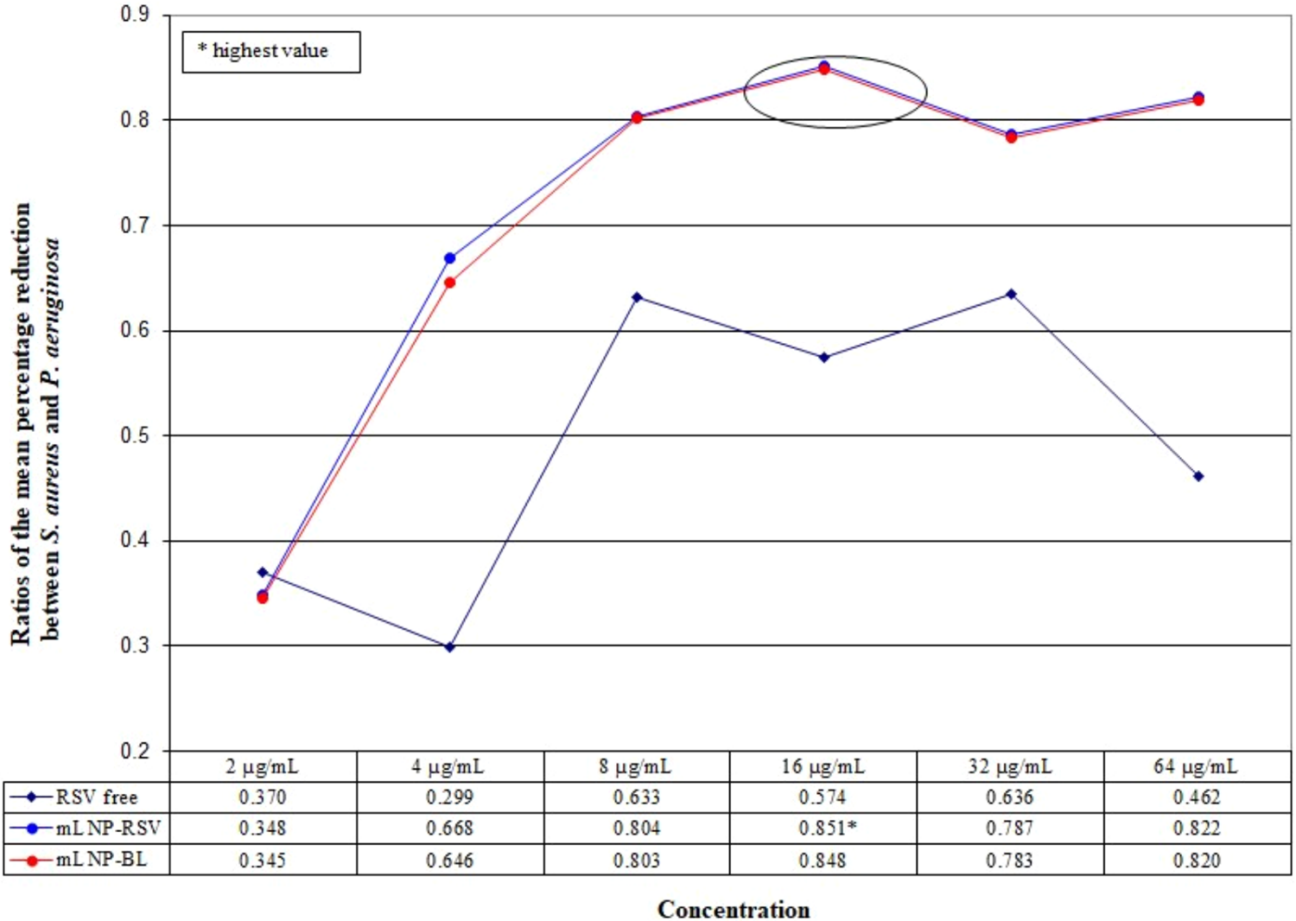
Ratios of the mean percentage reduction between *S. aureus* and *P. aeruginosa* at 2, 4, 8, 16, 32, and 64 μg/mL.

The treatment with 16 μg/mL RSV (either as a free solution or as an mLNP-RSV dispersion) was the most effective in preventing biofilm formation in the tested bacterial strain. Increasing the RSV concentration beyond this value did not lead to any additional enhancement of the inhibitory effect on biofilm formation; therefore, higher concentrations were excluded from subsequent analyses. A weak inhibitory effect on biofilm formation was also observed for mLNP-BL, which suggests a partial contribution of the lipid-based carrier to the overall antibiofilm activity, likely due to the presence of the functional excipients. However, this effect alone was not sufficient to warrant their inclusion in subsequent studies of clinical isolates.

In Table 4, we report for Biofilm Group, such as CG, free RSV, and mLNP-RSV, the means and medians for Global OD and Global category variables, and the percentages of each category (Absent, Weak, Moderate, and Strong), for *S. aureus* and *P. aeruginosa* isolates. Additionally, in the last column, a comparison among Biofilm Group was reported.

**Table 4:** Means and Medians of Global Category and Global OD Variables of *S. aureus* and *P. aeruginosa* Isolates in CG, Free RSV, and mLNP-RSV^a^,^b^

*S. aureus*				
	GC	free RSV	mLNP-RSV	statistical analysis among
variables/biofilm group	*n* = 50	*n* = 50	*n* = 50	GC, free RSV, and mLNP-RSV
*global OD*				
mean ± SD	0.36 ± 0.40	0.15 ± 0.08	0.10 ± 0.05	*p* < 0.0001* (Fr)
median (IQR)	0.24 (0.13–0.45)	0.12 (0.09–0.20)	0.084 (0.06–0.11)	• CG vs free RSV (median: 0.24 vs 0.12), *p* < 0.05* (W)
SEM	0.057	0.012	0.113	• CG vs mLNPs-RSV (median: 0.24 vs 0.084), *p* < 0.05* (W)
[min, max]	(0.06, 2,44)	(0.02, 0.41)	(0.02, 0.27)	• free RSV vs mLNP-RSV (median: 0.12 vs 0.084), *p* < 0.05* (W)
*global category*				*p* < 0.0001* (Fr)
mean ± SD	3.42 ± 0.73	2.88 ± 0.80	2.48 ± 0.89	• CG vs free RSV (median: 4 vs 3), *p* < 0.05* (W)
median (IQR)	4 (3–4)	3(2–3)	3(2–3)	• CG vs mLNP-RSV (median: 4 vs 3), *p* < 0.05* (W)
				• free RSV vs mLNP-RSV (mean rank: 1.94 vs 1.49), *p* < 0.05* (W)
*categories*				
absent	↓	3 (6%)	8 (16%)	free RSV and mLNP-RSV (16% vs 6%), *p* = 0.13 (M)
weak	7 (14%)	10 (20%)	15 (30%)	*p* = 0.10(Q)
moderate	15 (30%)	27(54%)	22 (44%)	*p* = 0.020*(Q)
				• CG vs free RSV (30% vs 54%), *p* < 0.05* (MRD)
strong	28 (56%)	10 (20%)	5 (10%)	*p* < 0.0001*(Q)
				• CG vs free RSV (56% vs 20%), *p* < 0.05* (MRD)
				• Free RSV vs mLNP-RSV (56% vs 10%), *p* < 0.05* (MRD)

*P. aeruginosa*				
				statistical analysis among
variables	CG	free RSV	mLNP-RSV	GC, free RSV, and mLNP-RSV
*global OD*				*p* < 0.0001* (Fr)
mean ± SD	0.24 ± 0.20	0.10 ± 0.05	0.08 ± 0.06	• CG vs free RSV (median: 0.18 vs 0.09), *p* < 0.05* (W)
median (IRQ)	0.18 (0.12–0.30)	0.09 (0.08–0.11)	0.077 (0.04–0.11)	• CG vs mLNP-RSV (median: 0.18 vs 0.077), *p* < 0.05* (W)
SEM	0.0028	0.007	0.008	• free RSV vs mLNP-RSV (median: 0.09 vs 0.077), *p* < 0.05* (W)
[min, max]	(0.04, 1.14)	(0.03, 0.26)	(0.01, 0.33)	
*global category*				*p* < 0.0001* (Fr)
mean ± SD	3.10 ± 0.86	2.28 ± 0.81	2.02 ± 0.89	• CG vs free RSV (median: 3 vs 2), *p* < 0.05* (W)
median (IRQ)	3 (2–4)	2(2 −3)	2(1–3)	• CG vs mLNP-RSV (median: 3 vs 2), *p* < 0.05* (W)
				• free RSV vs mLNP-RSV (mean rank: 1.76 vs 1.48), *p* < 0.05* (W)
*categories*				
absent	↓	10 (20%)	17 (34%)	free RSV vs mLNP-RSV (20% vs 34%), *p* = 0.0156* (M)
weak	16(32%)	17 (34%)	17 (34%)	*p* = 0.97(Q)
moderate	13 (26%)	22(44%)	15 (30%)	*p* = 0.06(Q)
strong	21 (42%)	1 (2%)	1 (2%)	*p* < 0.0001*(Q)
				• CG vs free RSV (42% vs 2%), *p* < 0.05* (MRD)
				• free RSV vs mLNP-RSV (42% vs 4%), *p* < 0.05* (MRD)

a IRQ = Interquartile range, SD = standard deviation; SEM = standard error of the mean; * = significant test; W = Wilcoxon test; Q= Cochran’s Q test; MRD = Minimum Required Differences method with Bonferroni post hoc Q test; Fr= Friedman ANOVA test; W = Wilcoxon test post hoc Friedman ANOVA test; M = McNemar’s exact test

b Additionally in the last column, the comparison among Biofilm groups was reported

The Global OD variable rejected the normality hypothesis for all cases. For *S. aureus*, the Global OD values of CG were significantly greater than those of the free RSV (median: 0.24 vs 0.12) and mLNP-RSV (median: 0.24 vs 0.084) groups, and the Global OD values of the free RSV group were significantly greater than those of the mLNP-RSV group (median: 0.12 vs 0.084). Analogous results were obtained by considering the Global category variable. Furthermore, the category “Absent” was only tested between free RSV and mLNP-RSV. In this case, no significant difference was observed between free RSV and mLNP-RSV groups (16% vs 6%, *p* = 0.13). “Moderate” was significantly more frequent in mLNP-RSV than in CG (54% vs 30%), while “Strong” category was more frequent in CG than free RSV (56% vs 20%) and mLNP-RSV (56% vs 10%).

Regarding the experiments performed against *P. aeruginosa*, the Global OD variable evaluation rejected the normality hypothesis for all cases. Moreover, the results obtained in terms of the Global OD variable and Global category were similar to those recorded for the *S. aureus* experiments.

Again, the category “Absent” was only tested between free RSV and mLNP-RSV. Particularly, the mLNP-RSV group highlighted a significantly more frequent “Absent” category than the free RSV one (34% vs 20%, p = 0.0156). For “Moderate” and “Weak”, no significant differences were observed among all 3 groups, while the “Strong” category was more frequent CG than free RSV (42% vs 4%) and mLNP-RSV (42% vs 2%) groups.

A comparative analysis of the *S. aureus* and *P. aeruginosa* experiments is reported in Table 5.

**Table 5:** Comparison between *S. aureus* and *P. aeruginosa* Experiments in Terms of Group about Global Category and Global OD Variables of GC, Free RSV, and mLNP-RSV^a^

*S. aureus* vs *P. aeruginosa*	
biofilm groups	statistical analysis between same biofilm groups
**CG**	
global OD	median: 0.24 vs 0.18, *p* = 0.15 (MW)
global category	median: 4 vs 3, *p* = 0.062 (MW)
absent	—-
weak	14% vs 32%, *p* = 0.0325* (C)
moderate	30% vs 26%, *p* = 0.65 (C)
strong	56% vs 42%, *p* = 0.16 (C)
**free RSV**	
global OD	median: 0.12 vs 0.09, *p* = 0.0016* (MW)
global category	median: 3 vs 2, *p* = 0.0005* (MW)
absent	6% vs 20%, *p* = 0.0374* (C)
weak	20% vs 34%, *p* = 0.11 (C)
moderate	54% vs 44%, *p* = 0.32 (C)
strong	20% vs 2%, *p* = 0.004* (C)
**mLNP-RSV**	
global OD	median: 0.084 vs 0.077, *p* = 0.16 (MW)
global category	median: 3 vs 2, *p* = 0.0118* (MW)
absent	16% vs 34%, *p* = 0.0377* (C)
weak	30% vs 34%, *p* = 0.67 (C)
moderate	44% vs 30%, *p* = 0.15 (C)
strong	10% vs 2%, *p* = 0.21(F)

a * = significant test; C = chi-square test; MW = test; *F* = Fisher’s exact test.

In both sets of experiments, the comparison between the CGs highlighted no significant differences in terms of Global OD and Global category variables. Instead, for the free RSV group, both the Global OD and the Global category gave significantly greater scores for *S. aureus* than for *P. aeruginosa* group (Median: 0.12 vs 0.09, *p* = 0.0016; Median: 3 vs 2, *p* = 0.0118; respectively). Finally, for the mLNP-RSV group, only the Global category resulted in a significantly greater score for *S. aureus* than for *P. aeruginosa* (Median: 3 vs 2, *p* = 0.0118).

Going into detail, as reported in Table 5, in the case of biofilm without treatment, the category “Weak” was more frequent for *P. aeruginosa* than *S. aureus*. Moreover, for the free RSV group, the category “Absent” was significantly more frequent in *P. aeruginosa* than *S. aureus* (20% vs 6%, p = 0.0374), vice versa for the category “Strong” (2% vs 20%, *p* = 0.004).

Also, for the mLNP-RSV group, the category “Absent” was more frequent in *P. aeruginosa* than *S. aureus* (34% vs 16%, p = 0.0377).

As shown in Table 6, all strains (100%) of *S. aureus* were positive for *icaA*, *icaD*, and *clfA* genes, while 29 (58%), 49 (98%), and 7 (14%) strains were positive for *clfB*, *fnbpA*, and *fnbpB* genes.

**Table 6:** Presence of Genes Associated with Biofilm Production in *S. aureus*, Stratified According to Observed Biofilm Phenotypes (“Strong,” “Moderate,” “Weak”, “Absent”)^a^

*S. aureus*: biofilm CG						
gene	PCR detect	absent	weak	moderate	strong	comparison among categories: *p*-value (test)
icaA	100% (50) +	0.0% (0)	14% (7)	30% (15)	56% (28)	1.0 (F)
	0.0% (0) -	0.0% (0)	0.0% (0)	0.0% (0)	0.0% (0)	
icaD	100% (50) +	0.0% (0)	14% (7)	30% (15)	56% (28)	1.0 (F)
	0.0% (0) -	0.0% (0)	0.0% (0)	0.0% (0)	0.0% (0)	
clfA	100% (50) +	0.0% (0)	14% (7)	30% (15)	56% (28)	1.0 (F)
	0.0% (0) -	0.0% (0)	0.0% (0)	0.0% (0)	0.0% (0)	
clfB	58% (29) +	0.0% (0)	8% (4)	16% (8)	34% (17)	0.92 (F)
	42% (21) -	0.0% (0)	6% (3)	14% (7)	22% (11)	
fnbpA	98% (49) +	0.0% (0)	14% (7)	30% (15)	54% (27)	1.0 (F)
	2% (1) -	0.0% (0)	0.0% (0)	0.0% (0)	2% (1)	
fnbpB	14% (7) +	0.0% (0)	4% (2)	6% (3)	4% (2)	0.21 (F)
	86% (43) -	0.0% (0)	10% (5)	24% (12)	52% (26)	

*S. aureus* *:* biofilm free RSV						
gene	PCR detect	absent	weak	moderate	strong	comparison among categories: *p*-value (test)
*icaA*	100% (50) +	6% (3)	20% (10)	54% (27)	20% (10)	1.0 (F)
	0.0% (0) -	0.0% (0)	0.0% (0)	0.0% (0)	0.0% (0)	
*icaD*	100% (50) +	6% (3)	20% (10)	54% (27)	20% (10)	1.0 (F)
	0.0% (0) -	0.0% (0)	0.0% (0)	0.0% (0)	0.0% (0)	
*clfA*	100% (50) +	6% (3)	20% (10)	54% (27)	20% (10)	1.0 (F)
	0.0% (0) -	0.0% (0)	0.0% (0)	0.0% (0)	0.0% (0)	
*clfB*	58% (29) +	2% (1)	14% (7)	40% (20)	2% (1)	0.0019* (F)
	42% (21) -	4% (2)	6% (3)	14% (7)	18% (9)	moderate (+)**, *p* = 0.0126 (Z)
						strong (−)**, *p* = 0.0006 (Z)
*fnbpA*	98% (49) +	6% (3)	20% (10)	54% (27)	18% (9)	0.46 (F)
	2% (1) -	0.0% (0)	0.0% (0)	0.0% (0)	2% (1)	
*fnbpB*	14% (7) +	0.0% (0)	4% (2)	10% (5)	0.0% (0)	0.57 (F)
	86% (43) -	6% (3)	16% (8)	44% (22)	20% (10)	

*S. aureus* *:* biofilm mLNP-RSV						
gene	PCR detect	absent	weak	moderate	strong	comparison among categories: *p*-value (test)
*icaA*	100% (50) +	16% (8)	30% (15)	44% (22)	10% (5)	1.0 (F)
	0.0% (0) -	0.0% (0)	0.0% (0)	0.0% (0)	0.0% (0)	
*icaD*	100% (50) +	16% (8)	30% (15)	44% (22)	10% (5)	1.0 (F)
	0.0% (0) -	0.0% (0)	0.0% (0)	0.0% (0)	0.0% (0)	
*clfA*	100% (50) +	16% (8)	30% (15)	44% (22)	10% (5)	1.0 (F)
	0.0% (0) -	0.0% (0)	0.0% (0)	0.0% (0)	0.0% (0)	
*clfB*,	58% (29) +	6% (3)	20% (10)	30% (15)	2% (1)	0.14 (F)
	42% (21) -	10% (5)	10% (5)	14% (7)	8% (4)	
*fnbpA*	98% (49) +	16% (8)	30% (15)	42% (21)	10% (5)	1.0 (F)
	2% (1) -	0.0% (0)	0.0% (0)	2% (1)	0.0% (0)	
*fnbpB*	14% (7) +	0.0% (0)	8% (4)	6% (3)	0.0% (0)	0.37 (F)
	86% (43) -	16% (8)	22% (11)	38% (19)	10% (5)	

a * = significant test; ** = more frequent modality; *F* = Fisher’s exact test; Z = z-test.

Table 6 shows a significant relationship between *clfB* gene and biofilm phenotypes (*p* = 0.0019) for Biofilm Free RSV. Particularly, the most frequent biofilm phenotypes were Moderate (+) (40%, *p* = 0.0126) and Strong (−) (18%, *p* = 0.0006).

Table 7 shows that all *P. aeruginosa* strains (100%) were positive for the *algD* gene, while the genes *pelF* and *pslD* were present in 44 (88%) and 47 (94%) strains, respectively.

**Table 7:** Presence of Genes Associated with Biofilm Production in *P. aeruginosa*, Stratified According to Observed Biofilm Phenotypes (“Strong,” “Moderate,” “Weak”, “Absent”)^a^

*P. aeruginosa*: biofilm CG						
gene	PCR detect	absent	weak	moderate	strong	comparison among categories: *p*-value (test)
*algD*	100% (50) +	0.0% (0)	32% (16)	26% (13)	42% (21)	1.0 (F)
	0.0% (0) –	0.0% (0)	0.0% (0)	0.0% (0)	0.0% (0)	
*pelF*	88% (44) +	0.0% (0)	32% (16)	20% (10)	36% (18)	0.14 (F)
	12% (6) –	0.0% (0)	0.0% (0)	6% (3)	6% (3)	
*pslD*	94% (47) +	0.0% (0)	32% (16)	24£ (12)	38% (19)	0.61 (F)
	6% (3) –	0.0% (0)	0.0% (0)	2% (1)	4% (2)	

*P. aeruginosa* *:* biofilm free RSV						
gene	PCR detect	absent	weak	moderate	strong	comparison among categories: *p*-value (test)
*algD*	100% (50) +	20% (10)	34% (17)	44% (22)	2% (1)	1.0 (F)
	0.0% (0) –	0.0% (0)	0.0% (0)	0.0% (0)	0.0% (0)	
*pelF*	88% (44) +	20% (10)	32% (16)	36% (18)	0.0% (0)	0.067 (F)
	12% (6) –	0.0% (0)	2% (1)	8% (4)	2% (1)	
*pslD*	94% (47) +	20% (10)	34% (17)	38% (19)	2% (1)	0.29 (F)
	6% (3) –	0.0% (0)	0.0% (0)	6% (3)	0.0% (0)	

*P. aeruginosa* *:* biofilm mLNP-RSV						
gene	PCR detect	absent	weak	moderate	strong	comparison among categories: *p*-value (test)
*algD*	100% (50) +	34% (17)	34% (17)	30% (15)	2% (1)	1.0 (F)
	0.0% (0) –	0.0% (0)	0.0% (0)	0.0% (0)	0.0% (0)	
*pelF*	88% (44) +	34% (17)	28% (14)	26% (13)	0.0% (0)	0.24 (F)
	12% (6) –	0.0% (0)	6% (3)	4% (2)	2% (1)	
*pslD*	94% (47) +	34% (17)	32% (16)	26% (13)	2% (1)	0.62 (F)
	6% (3) –	0.0% (0)	2% (1)	4% (2)	0.0% (0)	

a * = significant test; ** = more frequent modality; *F* = Fisher’s exact test; Z = z-test.

Table 7 shows no significant differences between genes associated with biofilm production in *P. aeruginosa* and biofilm phenotypes.

In *S. aureus*, biofilm production was specifically associated only with the presence of the *clfB* gene, which encodes an adhesin capable of binding fibrinogen,^26^ particularly when moderately producing isolates are considered. It is instead close to significance (0.14) when the microorganism is a high producer. In *P. aeruginosa*, biofilm production was of near-significance only in the presence of the *pelF* and *pslD* genes.

## 4 Discussion

Nowadays, antibiotic resistance represents one of the major global health problems due to its constant increase. As a consequence, there is a great need to find new compounds to overcome this issue. Moreover, by forming a biofilm, bacteria protect themselves from host defense, disinfectants, and antibiotics.^27^ The bacteria inside the biofilm are also much more resistant to antimicrobial agents than planktonic forms, since bacteria that are nonresistant to antimicrobial agents in any way can turn resistant after forming a biofilm. Over the years, plant extracts, essential oils, small antimicrobial peptides of animal origin, bacteriocins, and various groups of other plant-derived compounds have been demonstrated to possess antimicrobial and antibiofilm activities.^28^

Moreover, scientists are developing nanocarrier-based drug delivery systems to improve the effectiveness of plant-derived compounds, which often have poor water solubility and limited absorption. Nanostructured carriers like polymeric nanoparticles, liposomes, lipid-based nanoparticles, micelles, and nanoemulsions are currently explored to enhance the oral delivery, bioavailability, and safety of drugs.^29^ These nanomedicines help overcome challenges related to solubility, absorption, and toxicity of natural compounds, enhancing their antimicrobial and antibiofilm activities.^30,31^

As is known, *S. aureus* and *P. aeruginosa* are the two major pathogens that can cause biofilm-associated infections and thus the evaluation of novel drug delivery systems loaded with natural plant-based chemicals with biofilm-forming inhibitory ability should be focused on in vitro studies against these two main bacterial species^32^

The activity of RSV in inhibiting the biofilm and virulence factors of several bacteria has been widely reported. Moreover, the clinical use of RSV is currently restricted due to its disadvantageous physicochemical properties, poor water solubility, and extensive hepatic metabolism after oral administration, leading to handling difficulties and low bioavailability. When RSV was delivered using multicomponent lipid nanoparticles (mLNPs), it exhibited enhanced fibroblast proliferation and migration capabilities, as well as antibiofilm properties, even at extremely low doses, also demonstrating wound-healing potentialities.^16^ The choice of embedding RSV into the mLNPs is related to the main idea of developing an active DDS composed of functional lipid excipients, which could confer certain basic properties to the DDS itself. In this context, the use of functional lipids possessing scavenging and antimicrobial properties could boost the RSV effect. According to these considerations, the composition of the mLNP-RSV reported here is the following:

- Labrasol, which is the liquid lipid used to dissolve RSV,
- Glyceryl monostearate, which is the solid lipid used to give consistency and stability to the resulting DDS,
- 18-β-Glycyrrhetinic acid, which is a triterpenoid compound derived from licorice root and possesses immunomodulatory, antioxidant, antibacterial, and anti-inflammatory properties,^33^
- Menthol, which is a natural terpene well known for its antimicrobial properties.^34^

In the present study, we investigated the use of mLNP-RSV to allow a targeting action toward bacterial biofilm formation, compared to free RSV. We preliminarily evaluated the activity of mLNP-RSV formulation at different concentrations ranging from 2 to 64 μg/mL, on strains ATCC 12972 (*S. aureus*) and ATCC 27853 (*P. aeruginosa*) to establish the subinhibitory concentration with the best action to be used for further testing versus bacterial clinical strains. Several studies have shown that RSV can inhibit biofilm formation in multiple bacterial species. Significant antibiofilm effects have been reported in Gram-negative bacteria at concentrations of approximately 1–30 μg/mL, whereas Gram-positive bacteria (e.g., *Listeria* spp.) generally require higher concentrations. These values vary depending on the bacterial strain, experimental conditions, and analytical methods.^25,35^ In our study, in agreement with the current literature findings, 16 μg/mL was identified as the lowest tested concentration of RSV capable of reducing biofilm formation; however, the extent of this reduction differed between Gram-negative and Gram-positive bacteria.

Although promising, these findings should be considered preliminary, as they were obtained under specific in vitro experimental conditions. Nonetheless, this preliminary evidence provides a valuable starting point for the development of novel RSV-based antimicrobial strategies. In our study, the administration of free RSV 16 μg/mL inhibited the biofilm formation of 35.6% and 62.1% on the total biofilm mass for *S. aureus* and *P. aeruginosa*, respectively, while a higher percentage of inhibition was found for the mLNP-RSV group (administered in order to achieve the same RSV dose): 51.4% and 63.0%, respectively. These preliminary results suggest a potential enhancement of RSV activity by the proposed DDS, which might be related to the lipid composition of the nanoparticles and to an improved interaction between RSV and bacterial cells enabled by the nanodelivery strategy. This hypothesis is in line with the previous observation of a certain antibiofilm activity, also when treating the ATCC strains with mLNP-BL. Analyzing two analogous isolated strains, we observed not only a significant reduction of OD score between the biofilm, biofilm + free RSV, or + mLNP-RSV groups, but also a reduction in category. Furthermore, we observed a significant presence of isolates with the Strong category for the biofilm group and a significant presence of isolates with the Absent category for the biofilm + free RSV or + mLNP-RSV groups. These results indicate that the administration of mLNP-RSV was associated with a significant reduction in the presence of biofilm, especially in *P. aeruginosa* strains. Moreover, these findings indicate a potential advantage of a nanoparticle-based treatment rather than free RSV alone. In this regard, it should also be considered a key issue: to achieve the chosen RSV concentration to perform the free RSV experiments, it was mandatory to dissolve RSV in DMSO prior to diluting this RSV stock solution into the culture medium. The latter is due to the poor RSV solubility in aqueous media, which was effectively overcome by embedding it into the mLNPs. Indeed, although the statistical difference in the percentage of inhibition between free and mLNP-encapsulated RSV is minimal, this must be interpreted in a clinical context, where the poor solubility of free RSV prevents it from reaching comparable concentrations. Thus, the observed in vitro activity suggests that mLNP delivery enables RSV effectiveness.

The relationship between the presence of genes more frequently associated with biofilm production was also analyzed, which differed between the two strains. In accordance with the literature,^36^ this correlation appeared to be confirmed for the *clfB* gene in *S. aureus*. The absence of a clear correlation with the other genes suggests that biofilm production is influenced by additional genes and/or other regulatory or environmental factors. Given the exploratory nature of this study, these findings further highlight the need for additional investigations using a larger number of samples.

Another relevant point concerns the differences in activity observed between Gram-positive and Gram-negative strains, which we hypothesized to be at least in part related to their distinct cell wall compositions. In other words, our results are encouraging and support the hypothesis that the antibiotic/resveratrol combination could represent a promising strategy for inhibiting biofilm formation at early stages and for eradicating established biofilms at later stages. Indeed, as previously suggested by Wang et al., and considering the good results obtained, mLNP-RSV could potentially serve as an adjuvant to antibiotic therapy for the treatment of infection due to MDR *P. aeruginosa*.^37,38^

In addition, it can be hypothesized that the use of mLNP-RSV could enhance bioavailability and potentially reduce the rapid metabolism and elimination of RSV, thereby enabling more effective exploitation of its chemopreventive properties, including anti-inflammatory and cytoprotective activities. The antibiofilm activity of RSV has been reported to affect bacterial adhesion and enhance the efficacy of antimicrobial drugs. Based on its observed effects on microbial biofilms, RSV delivered via suitable nanocarriers could be employed to reduce microbial pathogenicity and potentiate antimicrobial activity. Moreover, the integration of RSV into innovative therapeutic strategies provides a potentially promising approach for both the prevention of biofilm-associated infections and the mitigation of antimicrobial resistance.

Considering the results obtained in this work, we suggest that RSV exhibits variable effects depending on the type of microorganism and the specific bacterial strains involved. These differences highlight the importance of tailoring therapeutic strategies to the microbial target. The use of multicomponent lipid nanoparticles (mLNPs) appears to enhance the stability and biological activity of RSV, offering a promising platform to support its translation into clinical practice.

Future investigations should therefore focus on optimizing nanocarrier formulations and assessing their efficacy in clinically relevant models to fully exploit the potential of RSV in combination therapies. Moreover, previous evidence has demonstrated the cytocompatibility of the mLNP-RSV system toward fibroblasts, along with its wound-healing properties.^16^ In a subsequent study, De Caro et al. (2025) extensively investigated the stabilization of the nanoparticle dispersion by lyophilization, aiming to obtain a versatile and easily handled powder.^15,16^ These findings, together with the results of the present study, support the potential consideration of this system as a promising antibiofilm platform with substantial translational potential. Its potential applications range from topical administration (e.g., post-extractive dental sockets and cutaneous applications) to surface functionalization of medical devices and materials, including catheters and titanium implants. Furthermore, such nanocarriers could be further engineered or functionalized to specifically interact with different pathogens or infection sites, thereby improving the targeted delivery and therapeutic efficacy.

Collectively, these considerations provide a rationale for further multidisciplinary studies to elucidate and expand the applicative potential of this novel nanoscale delivery system.

## 5 Limitations

This study represents the authors′ first investigation into this topic, and although the results are promising, several limitations should be acknowledged. First, the work was conducted on a limited number of clinical strains, and only a single fixed concentration of RSV was tested, based on preliminary studies with ATCC strains. Expanding both the panel of clinical isolates and the range of RSV concentrations would provide a more comprehensive assessment of the formulation’s efficacy.

Additional limitations include the lack of evaluation of the mLNP-BL formulation on clinical isolates and the inherent limitation of the crystal violet assay, which cannot distinguish between live and dead cells, potentially affecting biofilm quantification. Another limitation is the absence of in vivo concentration data after in situ administration, preventing a correlation between effective in vitro concentrations and those attainable at the target site in vivo.

Finally, a detailed mechanistic study of the interactions between the nanoparticles and the bacterial cells is lacking. Addressing this aspect will be the focus of future investigations to fully define the advantages of the proposed nanosystem.

## 6 Conclusion

Many studies of natural products as plant-derived extracts have been proposed during recent years for the treatment of biofilm-associated infections, currently difficult to manage due to the increase of resistance to antibiotics causing negative results of the available treatment options.^39^ The results reported here not only confirm the inhibitory effects on biofilm formation of RSV, already described by different authors, but also emphasize the efficacy of RSV when delivered through ad hoc designed multicomponent lipid nanoparticles, chosen as innovative and functional drug carriers. Indeed, as reported here, RSV has been shown to reduce the level of bacterial biofilm formation, supporting its potential use as an adjuvant in the treatment of infections caused by both Gram-negative and Gram-positive bacteria. However, the poor aqueous solubility of RSV poses a significant challenge both for in vitro evaluation and, even more critically, for its clinical administration. To overcome these issues, mLNP-RSV were previously designed, optimized, and characterized. Based on their favorable properties, these nanoparticles were evaluated to assess their antibiofilm activity and their ability to enhance RSV efficacy. The results suggested that RSV delivery via mLNP-RSV could lead to an improved antibiofilm effect compared with free RSV. These findings are preclinical in nature, and further studies are required to evaluate the activity of mLNP-RSV against additional clinical strains and its potential use in combination with conventional antimicrobial therapies.

## Supplementary Material



## Notes

Antibiotics profile of *P. aeruginosa* (Table S1) and antibiotics profile of *S. aureus* (Table S2) (PDF)
