# Supplementary material for: Multicomponent Lipid Nanoparticles as a Tool to Potentially Improve the Antibiofilm Activity of Resveratrol against MDR Gram-Positive and Gram-Negative Clinical Isolates
Source: ACS Omega. 2026 Apr 24;11(17):25277–88. doi: 10.1021/acsomega.5c12649 (PMC13150613; doi:10.1021/acsomega.5c12649)
Supplement: Supplementary file 1 [file ao5c12649_si_001.pdf]

Supplementary file

Multicomponent Lipid Nanoparticles as a Tool to Potentially Improve the Antibiofilm Activity of Resveratrol Against MDR Gram-Positive and Gram-Negative Clinical Isolates

Giulia Di Prima[1], Maria Rita Tricoli[2]†, Nicola Serra[3]†, Viviana De Caro[1]*, Ignazio Arrigo[2], Cecilia La Mantia[4], Paola Di Carlo[2], Orazia Diquattro[5], Anna Giammanco[2] and Teresa Fasciana[2]

[1] Department of Biological, Chemical and Pharmaceutical Sciences and Technologies, University of Palermo, Via Archirafi 32, 90123 Palermo, Italy viviana.decaro@unipa.it; giulia.diprima@unipa.it;

[2] Department of Health Promotion, Mother and Child Care, Internal Medicine and Medical Specialties G. D'Alessandro, University of Palermo, Piazza delle Cliniche, 2, 90127 Palermo, Italy, mariaritatricoli@gmail.com, ignazio.arrigo90@gmail.com, anna.giammanco@unipa.it, paola.dicarlo@unipa.it, teresa.fasciana@unipa.it.

[3]Audiology Unit, Department of Neuroscience, Reproductive Sciences and Dentistry, University of Naples Federico II, Via Pansini 5, 80131 Naples, Italy, nicola.serra@unina.it

[4] Department of Precision Medicine in Medical, Surgical and Critical Care (Me.Pre.C.C.), University of Palermo, Via Liborio Giuffrè, 5, 90127 Palermo, Italy. cecilia.lamantia@unipa.it.

[5] Laboratory of Microbiology, A. O. Ospedali Riuniti "Villa Sofia-Cervello", 90100 Palermo, Italy

* Correspondence: author: viviana.decaro@unipa.it

† These authors contributed equally to this work.

Table S1. Antibiotics profile of *P. aeruginosa*

Antibiotics profile of *P. aeruginosa*														
	**Antibiotics**													
**Strains**	**AN**	**ATM**	**FEP**	**CAZ**	**CAZ-AVI**	**CIP**	**CS**	**IPM**	**LVX**	**MEM**	**MEM7VAB**	**PIP**	**TZP**	**TM**
**P 1**	S		R	I		R	S	I	R		S	I	I	S
**P 2**	S	I	I	I	S	I	S	I	I	S	S		I	S
**P 3**	S		R	I		I	S	I	I		S	I	I	S
**P 4**	S	R	R	R	S	I	S	I	I	S	S		R	S
**P 5**	S	I	I	R	S	R	S	R		S	R		R	R
**P 6**	S		I	I	S	I	S	I	I		S		I	S
**P 7**	S	I	I	I	S	I	S	I	I	S	S		I	S
**P 8**	S	I	I	I	S	I	S	I	I	S	S		I	S
**P 9**	S	I	I	I	S	I	S	I	I	S	S	I		S
**P 10**	S	I	I	I	S	I	S	I	I	S	S	I		S
**P 11**	S	I	I	I	S	I	S	I	I	S	S	I		S
**P 12**	S	I	I	I	S	I	S	I	I	S	S		I	S
**P 13**	S	I	I	I	S	I	S	I	I	S	S		I	S
**P 14**	S	I	I	I	S	I	S	I	I	S	S		I	S
**P 15**	S	I	I	I	S	I	S	I	I	S	S		I	S
**P 16**	S		R	R	S	I	S	I	I	S			R	S
**P 17**	S		I	I	S	I	S	R	I		I	I		S
**P 18**	S		I	I	S	I	S	I	I		S		I	S
**P 19**	S		I	I	S	I	S	I	I		S		I	S
**P 20**	S		I	I	S	I	S	I	I		S		I	S
**P 21**	S		I	I	S	I	S	I	I				I	S
**P 22**	S	I	I	I	S	I	S	I	I	S	S		I	S
**P 23**														
**P 24**	S		I	I		I	S	I	I		S	I	I	S
**P 25**	S		I	I		I		I	I		S	I	I	S
**P 26**	S		R	R	S	R	S	R	R		R		R	S
**P 27**	S	R	I	I	S	R	S	I		S	I		I	S
**P 28**	S		I	I	S	I	S	I	I		S		I	S
**P 29**	R		R	R	S	R	S	R	R		R		R	R
**P 30**	S		R	R		I	S	I	I		S	R	R	S
**P 31**	S	I	I	I	S	I	S		I		S		I	S
**P 32**	S	I	I	I	S	I	S	I	I	S	S		I	S
**P 33**	S		R	R	S	I	S	I	I		S		R	S
**P 34**	S	R	R	R	R	R	S	I		S	S		R	R
**P 35**	S		I	I	S	I	S	I	I		S		I	S
**P 36**	S	I	I	I	S	I	S	I	I	S	S		I	S
**P 37**	S	I	I	I	S	I	S	I	I	S	S		I	S
**P 38**			I	I	S	R	S	R	R		S		I	S
**P 39**			I	I	S	I	S	I	I		S		I	S
**P 40**		R	R	R	R	I	S	R	R		R		S	
**P 41**			I	I	I		S	I	I		S	I	I	S
**P 42**			I	I	S	I	S	I	I		S	I	S	

**P 43**			I	I	S	I	S	I	I		S		I	S
**P 44**	S	R	I	R	S	I	S	I	I	S	S	R		S
**P 45**	S	R	I	R	S	I	S	I	I	S	S	R		S
**P 46**	S	R	I	R	S	I	S	I	I	S	S	R		S
**P 47**	S		I	I	S	I	S	I	I		S		I	S
**P 48**	S		I	I	S	I	S	I	I		S		I	S
**P 49**	S		I	I		I	S	I	I		S	I	I	S
**P 50**	S		I	I		I	S	I	I		S	I	I	S

AN – Amikacin, ATM – Aztreonam, FEP – Cefepime, CAZ – Ceftazidime, CAZ-AVI – ceftazidime/Avibactam, CIP – Ciprofloxacin, CS – Colistin, IPM – Imipenem, LVX – Levofloxacin, MEM – Meropenem, MEM7VAB – Meropenem/Vaborbactam, PIP – Piperacillin, TZP – Piperacillin/Tazobactam, TM – Tobramycin. S – Susceptible, I – Susceptible, increased exposure, R – Resistant.

Table S2. Antibiotics profile of *S. aureus*

Antibiotics profile of *S. aureus*																					
	**Antibiotics**																				
**Strains**	**FA**	**AM**	**CIP**	**CM**	**C**	**DAP**	**E**	**FOS**	**GM**	**LVX**	**LZD**	**MXF**	**MUP**	**OX**	**P**	**TEC**	**TE**	**TGC**	**TM**	**TMP**	**VA**
**S 1**	S		I	S		S	S	S	S		S	S	S	R	R	S	R	S		S	
**S 2**	S		I	S	S	S	S	S	S	I	S	S	S	R	R	S	R	S	S	S	S
**S 3**	S		R	R		S	R	S	S		S	R	S	R	R	S	S	S		S	S
**S 4**	S	R	I	S		S	S	S	S		S	S	S	S	R	S	S	S		S	S
**S 5**	S	R	R	S		S	S	S	S		S	R	S	S	R	S	R	S		S	S
**S 6**	S	R	R	S		S	S	S	S		S	R	S	S	R	S	S	S		S	S
**S 7**	S		I	R	S	S	R	S	S	I	S	S	S	S	R	S	S	S	S	S	S
**S 8**	S					S		S			S		S	R		S	S	S	S	S	S
**S 9**	S			S		S	S	R	S		S	R	S	R	R	S	S	S		S	S
**S 10**	S		I	R		S	R	S	S		S	S	S	R	R	S	R	S		S	S
**S 11**	S		R	R	S	S	R	R	S	R	S	R	S	R	R	S	S	S	S	S	S
**S 12**	S			R		S	R	S	S		S	R	S	R	R	S	S	S		S	S
**S 13**	S		I	R		S	R	S	S		S	S	S	R	R	S	S	S		S	S
**S 14**	S	R	I	S		S	S	S	S		S	S	S	R	R	S	R	S		S	S
**S 15**	S		R	R		S	R	S	S		S	R	S	R	R	S	S	S		S	S
**S 16**	S		I	R		S	R	S	S		S	S	S	R	R	S	R	S		S	S
**S 17**	S		R	R		S	R	S	S		S	R	S	R	R	S	S	S		S	S
**S 18**	S		R	R		S	R	S	S		S	R	S	R	R	S	S	S		S	S
**S 19**	S		R	S		S	S	R	S		S	R	S	R	R	S	S	S		S	S
**S 20**	S		I	R		S	R	S	S		S	S	S	R	R	S	R	S		S	S
**S 21**	S		R	R		S	R	S	R		S	R	S	R	R	S	S	S		S	S
**S 22**	R		R	R	S	R	I	R	R	R	S	R	I	R	R	S	S	S	R	S	S
**S 23**	S		I	R		S	R	S	S		S	S	S	R	R	S	S	S		S	S
**S 24**	S		R	S	S	S	S	S	S	R	S	R	S	R	R	S	S	S	S	S	S
**S 25**	S		R	R		S	R	S	S		S	R	S	R	R	S	S	S		S	S
**S 26**	R		I	S	S	S	S	S	R	I	S	S	S	R	R	S	R	S	R	S	S
**S 27**	S		R	R		S	R	S	S		S	R	S	R	R	S	S	S		S	S
**S 28**	S		S	S		S	S	S	S	S	S	S	S	S	R	S	S	S		S	S
**S 29**	S		S	S		S	S	S	S		S	S	S	R	R	S	S	S		S	S
**S 30**	S		S	S		S	S	S	S		S	S	S	R	R	S	S	S		S	S
**S 31**	S		R	S		S	S	R	S		S	R	S	R	R	S	S	S		S	S
**S 32**	R		R	R		S	R	S	R		S	R	S	R	R	S	S	S		R	S
**S 33**	S	R	R	R		S	R	S	S		S	R	R	S	R	S	S	R		S	S
**S 34**	S	R	I	S		S	S	S	R		S	S	S	S	R	S	S	S		S	S
**S 35**	S		I	S	S	S	S	S	S	I	S	S	S	S	R	S	S	S		S	S
**S 36**	S		R	S	S	S	S	S	S	R	S	R	S	S	S	S	S	S		S	S
**S 37**	S	R	I	S		S	S	S	S		S	S	S	S	R	S	S	S		S	S
**S 38**	R	R	I	S		S	S	S	S		S	S	S	S	R	S	S	S		S	S
**S 39**	S	R	R	S		S	S	S	S		S	R	S	S	R	S	S	S		S	S
**S 40**	S		I	S	S	S	S	S	S	I	S	S	S	S	R	S	S	S	S	S	S
**S 41**	S		I	S	S	S	S	S	S	I	S	S	S	S	R	S	S	S	S	S	S
**S 42**	S	R	I	S		S	S	S	S		S	S	S	S	R	S	S	S		S	S
**S 43**	S	R	I	R		S	R	S	S		S	S	S	S	R	S	S	S		S	S
**S 44**	S	R	R	R	S	S	R	S	R	R	S	R	S	S	R	S	S	S	R	I	S
**S 45**	S		I	R		S	R	S	S		S	S	S	R	R	S	R	S		S	S
**S 46**	S	R	I	S		S	S	S	S		S	S	S	S	R	S	S	S		S	S
**S 47**	S	R	S	S		S	S	R	S		S	R	S	R	R	S	S	S		S	S
**S 48**	S	R	I	R		S	R	S	S		S	S	S	S	R	S	S	S		S	S
**S 49**	S	R	I	S		S	S	S	S		S	S	S	S	R	S	S	S		S	S
**S 50**	S	R	I	S		S	S	S	R		S	S	S	S	R	S	S	S		S	S

FA – Fusidic acid, AM – Ampicillin, CIP – Ciprofloxacin, CM – Clindamycin, C – Chloramphenicol, DAP – Daptomycin, E – Erythromycin, FOS – Fosfomycin, GM – Gentamicin, LVX – Levofloxacin, LZD – Linezolid, MXF – Moxifloxacin, MUP – Mupirocin, OX – Oxacillin, P – Penicillin, TEC – Teicoplanin, TE – Tetracycline, TGC – Tigecycline, TM – Tobramycin, TMP – Trimethoprim, VA – Vancomycin. S – Susceptible, I – Susceptible, increased exposure, R – Resistant.
